# Relationships between emotional labor, job burnout, and emotional intelligence: an analysis combining meta-analysis and structural equation modeling

**DOI:** 10.1186/s40359-024-02167-w

**Published:** 2024-11-18

**Authors:** Yin-Che Chen, Zhi-Ling Huang, Hui-Chuang Chu

**Affiliations:** Department of Educational Psychology and Counseling, National Tsing Hua University, 521 Nanda Rd, Hsinchu City, Taiwan

**Keywords:** Emotional labor, Job burnout, Emotional intelligence, Meta-analysis, Structural equation modeling

## Abstract

The present study adopted a meta-analysis design that incorporated structural equation modeling to explore the relationships between emotional labor (EL), job burnout (JB), and emotional intelligence (EI), and enable model validation. The results revealed that EL and JB were significantly and positively correlated, that EI was significantly and positively correlated with EL, and that EI was significantly and negatively correlated with JB. The SEM parameter estimation values were all positive, reaching the level of significance and meeting the basic fit criteria. The total effect size of EL on JB was 0.289, which was equal to the sum of the direct and indirect effect sizes (0.371–0.082). This result indicated that EL affected JB through EI, validating the presence of a moderating effect. Finally, the results were discussed, and practical suggestions were proposed.

**Supplementary Information:**

The online version contains supplementary material available at 10.1186/s40359-024-02167-w.

## Introduction

With the thriving development of organizational psychology and the increasing corporate emphasis on the emotional well-being of employees and their physiological and psychological health, scholars have increasingly explored emotional cultures, individual affective expressions, and work results in an organizational context to develop concrete and effective suggestions for corporate operations and management. Hochschild introduced the concept of emotional labor (EL) and the use of the organizational emotion norms as a standard for evaluating employee work performance [1],. EL is regarded as a form of behavioral performance that aligns with emotion norms, indicating that emotions are an aspect of work. However, excessive EL or a dissonance between emotions norms and an individual’s personal emotions may induce stress or lead to negative results, such as perceived job burnout (JB), changes in work attitude, and a decline in service performance [2, 3].

Conservation of resources theory posits that when employees adhere to organizational emotion norms and adjust their emotions accordingly, they may expend considerable resources. Excessive emotional demands lead to JB, and a high EL may further exhaust an individual’s emotional resources, thereby causing them to experience various negative situations, including emotional exhaustion, depersonalization, and reduced personal accomplishment. These situations are regarded as the standard characteristics of JB [4]. Thus, if an organization intends to prevent its employees from experiencing the negative effects of EL, the emotional abilities of its employees is a key factor to consider. In an organization, individuals with higher emotional intelligence (EI) are more adept at adjusting their emotions to environmental demands related to EL [5]. Salovey and Mayerasserted that EI is a form of social intelligence that enables an individual to understand and manage interpersonal relationships [6]. In addition to its role in influence an individual’s emotional management and behavior, EI represents an individual’s ability to control their emotions and cope with frustrations when they encounter challenging events. The present study explored the model architecture underlying the associations among EL, JB, and EI to determine the relationships between specific variables. A review of studies conducted in Taiwan and other Asian countries was conducted to integrate the model and summarize generalizable results that are relevant to organizations.

Through the collection and collation of relevant literature, the present study determined that most studies related to EL, JB, and EI adopted a quantitative questionnaire survey design. Given the diverse outcomes reported in the literature, the present study adopted a meta-analysis research design and an integrated perspective to develop a theoretical model. Structural equation modeling (SEM) was used for validation, and a research model that combined meta-analysis and SEM (referred to as the “MASEM model”) was employed. Numerous scholars have adopted this model for research. Meta-analyses can overcome the disadvantages of traditional literature review methods by using statistical models to facilitate systematic integration and comments, thereby providing a focused and integrated architecture for studying specific topics. The meta-analysis model can be used to obtain objective quantitative information, assess the directions of the effects between variables, and determine the corresponding effect sizes [7]. Furthermore, the emerging MASEM method can validate the relevance between variables and correlation matrices by examining a proposed theoretical model and assessing its model fit [8]. Thus, the present study employed this research method to clarify and integrate literature findings and further explore the associations among EL, JB, and EI. The results and theoretical contribution of the present study are discussed, and practical suggestions are provided.

## Literature review

### Emotional labor

The concept of EL was first proposed by Hochschild, who defined EL as a process by which employees control and adjust their emotions during interactions with customers in the workplace to meet the normative demands of their organization with respect to personal emotional and behavioral expressions [1]. This process enables the integration of smiles, emotions, and affective relationships into the products or services sold. EL can be classified as surface and deep acting on the basis of an individual’s acting status. Surface acting involves an individual receiving instructions regarding emotional regulation without changing their thoughts through internalization. An individual who engages in surface acting adheres to organizational emotion norms and exhibit the corresponding attitudes. Deep acting occurs when an individual not only conforms to emotion norms but also internalize such norms and adjust their emotions such that they can meet organizational demands. In the Chinese language, various terms can be used to convey the concept of EL. However, scholars have extended the work of Hochschildby focusing on the active emotional regulation of employees as well as the management and training implemented by organizations and leaders to enable their employees to meet organizational emotional demands. Most subsequent scholars have adopted said definition of EL for further extension and elaboration [1]. Scholars worldwide have identified as EL as a service or a type of product on the basis of Hochschild’s finding regarding the positive and negative effects of EL on organizational development [1].

### Job burnout

The concept of burnout was first proposed by Freudenberger, who argued that burnout is not just a work-related status but one that an individual who is not affiliated with a unit may develop when they experience excessive demands on their resources, resulting in feelings of failure, weariness, and exhaustion [9]. Freudenberger contended that the loss of charisma by a leader is the primary antecedent of burnout and that when a leader begins to doubt their ability to competently manage their subordinates, their subordinates may experience disappointment or develop low expectations toward their leader [9]. Subsequently, the leader places excessive demands on the capabilities or resources of their subordinates to recover their charisma, thereby causing burnout and various forms of physiological and psychological discomfort. Maslach and Jacksoncontended that when employees experience increased levels of JB, they may become emotionally exhausted [10]. Excessive emotional exhaustion may reduce the job dedication of employees and have psychological effects. In addition, the employees may treat customers as an inconvenience when they are providing services and switch from active to negative attitudes or even adopt depersonalized modes. The employees tend to engage in negative self-evaluation, expressing considerable dissatisfaction with themselves and their jobs with respect to customer interactions. Thus, Maslach and Jacksondivided the aforementioned processes into three dimensions for measuring JB, namely, emotional exhaustion, depersonalization, and personal accomplishment, and they developed the Maslach Burnout Inventory (MBI) for assessing job burnout, which has been extensively applied by researchers [10].

### Emotional intelligence

EI can be explored from the perspectives of emotion and intelligence. Salovey and Mayer defined emotions as a series of organized responses related to physiology, cognition, motive, and experience [6]. Compared with moods, emotions have a shorter duration and are more intense. Emotions function as the positive or negative responses of an individual to internal and external events. Wechsler referred to intelligence as the comprehensive ability of an individual to respond appropriately to external environments by engaging in targeted thinking and rational decision-making [11]. Salovey and Mayer defined EI as a form of social intelligence that enables an individual to understand and manage interpersonal relationships [6]. This ability enables individuals to quickly classify their perceptions of life as emotions. In addition to facilitating internal emotional management, EI enables an individual to monitor the emotions of others and create an interactive atmosphere. This aspect of EI can be applied to predict future behavior. Salovey and Mayer proposed a conceptualized framework for using EI to evaluate emotions, the expression of personal emotions, the regulation of emotions, the application of emotions for flexible planning, the development of creative ideas, the transition of attention, and the generation of motivation [6]. In addition, they argued that the aforementioned concepts allow emotions to fulfill positive functions. Specifically, compared with the expression of simple individual emotion, EI emphasizes identifying personal emotions and those of others as a basis for regulating and solving problems.

### Relationships between emotional labor, job burnout, and emotional intelligence

#### Relationship between emotional labor and job burnout

A review of EL- and JB-related studies revealed that surface acting involves behavioral performance that does not involve internal thoughts and easily leads to emotional dissonance an individuals, causing them to experience psychological stress and possibly emotional exhaustion. When an employee endeavors to adhere to organizational emotion norms and realizes that such norms contradict their personal thoughts, they tend to engage in surface acting, which places an excessive demand on their emotional resources, resulting in the development of JB [12].

EL can easily occur in occupations that require frequent interactions with customers. To maintain emotional bonds with customers, organizations place excessive demands on their employees’ emotional resources, which tends to cause job stress and lead to JB. When EL is implemented by organizations to control the emotions of their employees, emotion management becomes a method for these employees to adhere to organizational norms or pursue organizational goals. In the service industry, employees must often interact with customers. To achieve the goals that are expected of them, employees must often adapt their emotions when they engage in EL [13]. Numerous scholars investigated frontline service providers as research participants and reported that differences between individual EL and organizational emotional regulation tend to cause emotional dissonance among employees. In addition, emotional dissonance may cause emotional exhaustion and depersonalization, both of which are dimensions of JB. Researchers have reported that individual EL is positively correlated with JB [4, 14].

Hsu and Lee [15] examined ground staff of an airline who experienced high EL in their study. The ground staff had to frequently interact with customers and adhere to strict emotion norms; thus, the study concluded that employees tend to develop JB when they are under the emotional control of their organizations. To alleviate such burnout, organizations can start by reducing the EL of their employees. Hsieh recruited hotel employees as participants and examined EL as a type of job demand [16]. Conducted on the basis of job resources theory, their study revealed an association between EL and JB. Specifically, if employees were willing to undergo internalized changes with respect to emotion norms, their adopted deep acting was negatively correlated with their JB. By contrast, if the employees adopted surface acting while providing services to customers in accordance with organizational rules, their surface acting was positively correlated with their JB. Chen and Wu examined the counter staff of department stores to explore the roles of emotional discrepancy and emotional efforts in EL [17]. Their results revealed that EL and JB are positively correlated. Employees who experience EL for a long period of time need to disguise their emotions or hide their true thoughts, which is a behavior that often depletes their emotional resources, reduces their personal accomplishment, and increases their JB.

In summary, the present study hypothesized that EL is significantly and positively correlated with JB. If employees comply with organizational emotion norms over a long period of time and experience emotional dissonance, JB may occur because of the discrepancy between their internal thoughts and external acting. Thus, the following hypothesis was established:

##### Hypothesis 1

EL is positively correlated with JB.

#### Relationship between emotional intelligence and emotional labor

Emotions were regarded as a display of individual behavior or a mental journey before Hochschild incorporated individual emotions and organizational norms into the concept of emotions and proposed the concept of EL, which considered the true emotions of employees, the emotional demands of enterprises, and actual dimensions of action transitions. Because the EL performed in an organization is regarded as a normative demand that can be managed and publicly displayed through various behavior, employees often express their emotional status through EL while interacting with others at work. Thus, employees who work in jobs that involve a high level of EL and possess high EI are regarded as employees who are highly competent at controlling and adapting their personal emotions to meet organizational demands. When employees accept emotion norms, they require more EI to adjust their personal feelings such that the depletion of their emotional and cognitive resources can be minimized [18].

To build enjoyable and comfortable environments in an era that emphasizes customer orientation, organizations are increasingly focusing on emotion norms without considering the emotional expression modes of individuals or the processes by which they interact with others. Numerous scholars have explored the relationship between EI and EL [19, 20]. Service sector studies have identified EI as a special ability on the basis that employees are required to implement the EL strategies of their organizations. Employees engage in deep or surface acting while interacting with customers. Under resources conservation theory, EI is an internal emotional resource of employees. If employees can adequately apply EI to perform the EL that is required of them at work, they are likely to adhere to organizational emotion norms. Through perceived organizational support, employees can supplement the emotional resources depleted by EL [21]. In addition, people with high EI can usually adapt their behavior in response to environmental changes, enabling them to adhere to emotion norms or display the emotions that are expected of them. Consequently, they EL load increases. Thus, in addition to the relationship between EI and EL, studies have often evaluated the effects of EI on the deep acting of employees and their EL loading; EI has also been studied as a moderating variable [22, 23].

EI not only affects the work of an employee but also facilitates the development of their interpersonal relationships if it is applied appropriately. Through behavioral performance, EI enables an employee to adjust or induce the emotional responses of others. Thus, an employee’s EI and EL acting behavior are positively correlated. If an employee has high EI, they can adequately implement emotional management depending on their emotional status to adjust their emotions through deep acting [24]. Yu (2020) conducted a study on the impact of emotional intelligence on emotional labor among employees of the Taiwan International Ports Corporation. The data analysis indicated that surface emotional intelligence could explain 23.2% of the variance in basic emotional expression, 6% in emotional diversity, 7% in deep emotional acting, 11% in surface emotional control, and 6.1% in interaction levels. The findings show that the higher the employees’ ability to perceive others’ emotions and regulate their own emotions, the greater the emotional labor stress related to basic emotional expression, deep emotional acting, surface emotional control, and frequent interactions required in their work. Additionally, employees with higher self-emotional assessment ability experienced less emotional labor stress from basic emotional expression [25]. Their results revealed that the employees with higher EI were more adept at applying, regulating, and controlling their personal emotions. The emotional changes that they underwent at work enabled them to perform EL involving emotional concealment, surface emotional control, and frequent interactions. These results suggest that partial variables that influence the relationship between EI and EL are significantly and positively correlated.

In summary, the present study hypothesized that EI and EL are significantly and positively correlated. Individuals with higher EI are more proficient at controlling their emotions and perceiving the emotions of others, thereby facilitating their rapid adaptation to environmental demands when they must adhere to organizational emotion norms. In the context of EL, they can adopt surface and deep acting to enhance the coherence and efficiency of their work and interpersonal relationships, thereby enabling higher levels of EL loading. Thus, the present study examined emotional loading in the context of EL and proposed the following hypothesis:

##### Hypothesis 2

EI is positively correlated with El.

#### Relationship between emotional intelligence and job burnout

Studies have indicated that emotional dissonance considerably affects JB. The ability to implement effective emotional control or regulation appears to play a crucial role in preventing affective disorders among employees at work [13]. Thus, enterprises should emphasize emotion-related dimensions and consider how the physiological and psychological status of their employees may affect their personal performance and the overall performance of an enterprise. Through their interpersonal relationships and the improvement of their individual EI, employees can appropriately manage their external behavioral performance and reduce negative work outcomes. Studies have reported that employee stressors can originate from both organizational and individual factors related to work. Excessive stress loading may exhaust individuals physiologically, psychologically, and spiritually, resulting in JB. Furthermore, because organizations are an external factor that is difficult to change and control, and that EI varies between individuals, Hung examined employees of small and medium-sized businesses, and reported that EI and JB are significantly and negatively correlated; specifically, among the various dimensions of EI, emotion regulation was most correlated with JB, followed by emotional expression and emotional awareness [26].

In addition, EI in the workplace can be classified into the three dimensions of emotional attention (i.e., the attention that an individual pays to the emotions of others), emotional understanding (i.e., the ability to understand, identify, and label the emotions of others), and emotional repair (i.e., the ability to regulate one’s emotions). A study reported that individuals with high EI can proficiently monitor the emotions of others, including the emotional responses of other personnel in the workplace. These individuals are also highly adept at understanding emotions and identifying the causes and effects of emotions, thereby reducing their work stress and alleviating various negative work effects and JB [27].

In their study, Liao and Song examined mass rapid transit drivers who were prone to experiencing high job stress [28]. Their study revealed that the appropriate application of EI by the drivers can effectively help them focus on their work and transform their stress into assistance, thereby helping them optimize their decision-making at work and to reduce their risk of developing JB. Their study also indicated that the EI and JB of the drivers were significantly and negatively correlated and that the application of EI reduced the development of JB when the drivers experienced job stress. In addition, their study employed the job requirements-resources model and asserted that employees with high EI can effectively allocate their job resources, quickly perceive emotions, and appropriately adjust their moods. These findings indicate that EI plays a role in moderating the relationship between job stress and JB.

Chong and Yen (2021) used hierarchical regression to examine the moderating effect of emotional intelligence on the relationship between emotional labor and job satisfaction. The data analysis indicated that emotional intelligence significantly moderates the relationship between interactional emotional labor and job satisfaction (beta = − 0.123, *p* = 0.045). For individuals with high emotional intelligence, the higher their interactional emotional labor, the lower their job satisfaction. However, for employees with low emotional intelligence, as interactional labor increases, their job satisfaction also increases [29]. Their study indicated that when individuals who are more adept at emotional regulation are also more cap Chong and Yen able of recognizing their personal emotions, addressing negative emotions, and, consequently, reducing their JB levels. Their results verified the presence of a negative relationship between EI and JB, implying that individuals with high EI can regulate their mindsets and emotions and are less likely to experience JB.

Burnout syndrome is defined as a general sense of psychological discomfort involving low levels of self-esteem, motivation, and occupational commitment. Burnout is a persistent state characterize by depressive and negative emotions. Work and occupational factors may contribute to the development of burnout. However, when an individual has higher EI, they can more capably resist JB by managing their emotions and emotional information. Through emotional clarity, organizations can ensure that their employees can perceive emotions and reduce tension and psychological dissonance. In addition, the attention dimension of EI can help employees focus on their emotions and those of others, thereby increasing their performance and concentration at work. Skillful emotional regulation allows for the conversion of negative emotions into positive ones, which can substantially reduce the development of JB [30]. In summary, the present study asserted that EI and JB are significantly and negatively correlated. When individuals have higher EI, they can more capably assess their personal emotional loading and adapt their emotions to regulate negative psychological perceptions and eliminate negative emotional thoughts. Such individuals are less likely to develop JB. Given these inferences, the present study proposed the following hypothesis:

##### Hypothesis 3

EI is negatively correlated with JB.

#### Relationships between emotional intelligence, emotional labor, and job burnout

Most studies that investigated EI, EL, and JB have reported that individuals in an organization who are more adept at emotional regulation or adaption have a greater influence on their EL and JB. Thus, in addition to exploring the associations between variables, numerous studies have examined the moderating effect of EI on the relationship between EL and JB and reported diverse results.

Taiwan-based studies that examined the moderating effect of EI on the relationship between EL and JB have identified significant correlations between EI and the other two variables. For example, a study reported that individual EI is positively correlated with workplace EL and that EI is negatively correlated with JB; however, the study asserted that the moderating effect of EI was different from the effect that was hypothesized [29].

Liu et al. contended that environmental and individual factors affect the relationship between EL and JB [31]. Individuals who are more adept at assessing emotions in the workplace can more competently address their negative psychological responses. Weng enrolled counseling psychologists as their study participants and reported that the psychologists who had to navigate the emotions of others while regulating their own personal emotions for an extended period of time in the workplace tended to perform increased EL and experience emotional exhaustion [32]. In this context, their emotional awareness, sense of comfort, and emotion utilization could be influencing factors. Although these studies examined the moderating effects of EI on the relationship between EL and JB, their results revealed the correlations between variables but did not support their hypothesis regarding moderating effects.

Studies conducted outside of Taiwan have also discussed the effect of EI on the relationship between EL and JB; however, their results differed from those of studies conducted in Taiwan. Hong and Lee examined a group of nurses and reported that when the nurses applied EI to fulfill their job requirements, they simultaneously engaged in EL and reduced their JB. Their results also indicated that EI mediates the relationship between EL and JB [33]. Lee and Chelladurai discussed the three dimensions of EL (i.e., surface acting, deep acting, and genuine expression), defining genuine expression as an active or conscious process involving changing emotions and asserting that surface acting and deep acting can be influenced by an individual’s EI, thereby affecting their level of emotional exhaustion [12]. Their study results verified that individuals who engage in more surface acting at work are more susceptible to emotional exhaustion because of the incongruity between their displayed emotions and genuine emotions. However, individuals with higher EI can more effectively regulate this process and reduce their JB. Scherer et al. argued that employees meet job requirements and adhere to emotion norms by engaging in EL, which is crucially influenced by the emotion management component of their individual EI [34]. EI not only facilitates effective emotional expression but also functions as a resource for coping with social interactions in the workplace, implementing constructive and efficient conflict management, and buffering against negative emotions. Consequently, EI helps an individual reduce their emotional exhaustion. Their results also indicated that individual EI effectively moderates the relationship between surface acting and JB.

In summary, on the basis of the results, hypotheses, and inferences of other studies, the present study hypothesized that an individual’s EI affects the relationship between EL and JB. When employees perform EL in accordance with the emotion-related rules of their organization, the effective application of personal EI to regulate emotions can reduce their risk of developing emotional exhaustion or JB. By contrast, individuals with low EI may experience more JB because of the EL they perform at work.

##### Hypothesis 4

EI moderates the relationship between EL and JB.

### Meta-analysis and structural equation modeling

Meta-analyses (MAs) have been widely conducted since the 1970s. An MA involves collecting data from diverse sources, including clinical trials, observational studies, and individual records. An MA is a tool for integrating and organizing large amounts of diverse data systematically. An MA involves primary and secondary data analysis, and it addresses research questions derived from original research studies and collates the findings from multiple studies. An MA is often referred to as analysis of analyses [35]. Numerous studies have been conducted to explore psychological outcomes. Although most of these studies have been sampled and systematically organized, the actual integration of such a great number of studies is rare. With the number of studies on popular topics continuing to increase, studies involving emerging and extensively studied issues require regular consolidation and summarization to assess their validity and outcomes. Therefore, to provide unbiased evaluations of existing evidence, scholars have started conducting quantitative MAs, which involve the quantification and integration of data from studies that covered similar topics but reported differing results. This research design enables scholars to understand various types of research pertaining to areas such as counseling, therapeutic effectiveness, and measurement outcomes [35, 36].

Structural equation modeling (SEM) has experienced rapid development since the 1970s as a technique for describing and estimating the linear relationships between variables. SEM involves latent and measured variables. Latent variables are hypothetical constructs that cannot be directly measured. Thus, researchers often use measured variables and study the directed and undirected linear relationships between these variables. The techniques applied include path analyses, factor analyses, analyses of variance, and regression analyses [37, 38]. In SEM analyses, a technique that can test the relationships in a set of variables enables the measurement of all the hypothesized relationships between the variables. Fit indices are employed to perform overall evaluations without the necessity of collecting raw data. These indices can be directly applied to a covariance or correlation matrix [38]. Researchers have employed SEM to conduct empirical research in various fields such as psychology, management, and other scientific disciplines. SEM enables researchers to examine the relationships between multiple concepts, assess the associations between variables on the basis of theoretical hypotheses, explore mediating effects, and evaluate the influence of multiple variables [39]. MacCallum and Austinconducted a synthesis of existing studies and discovered that SEM was commonly employed in fields such as applied psychology, organizational behavior, organizational psychology, and personnel psychology. Various studies have also adopted observational and experimental research designs [37].

## Research methods

### Research process

This study focuses on emotional labor, job burnout, and emotional intelligence as the main research themes. Initially, the direction of the study was primarily oriented towards the variable of “emotional labor,” as it has been extensively researched by scholars both domestically and internationally. Emotional labor has already established a stable and mature development in the domestic context, supported by a rich foundation of practical and theoretical knowledge. Subsequently, a review of the literature related to emotional labor was conducted to consolidate other frequently explored variables, serving as a reference to establish the study’s theme. Since this research employs integrative analysis as one of its primary research methods, it aims to identify theoretical models from past studies and to reexamine them as part of the research objectives for future inquiries. Subsequently, the co-authors conducted a compliance check for the inclusion of data and assessed the risk of research bias.

### Scope of literature

**Fig. 1:**
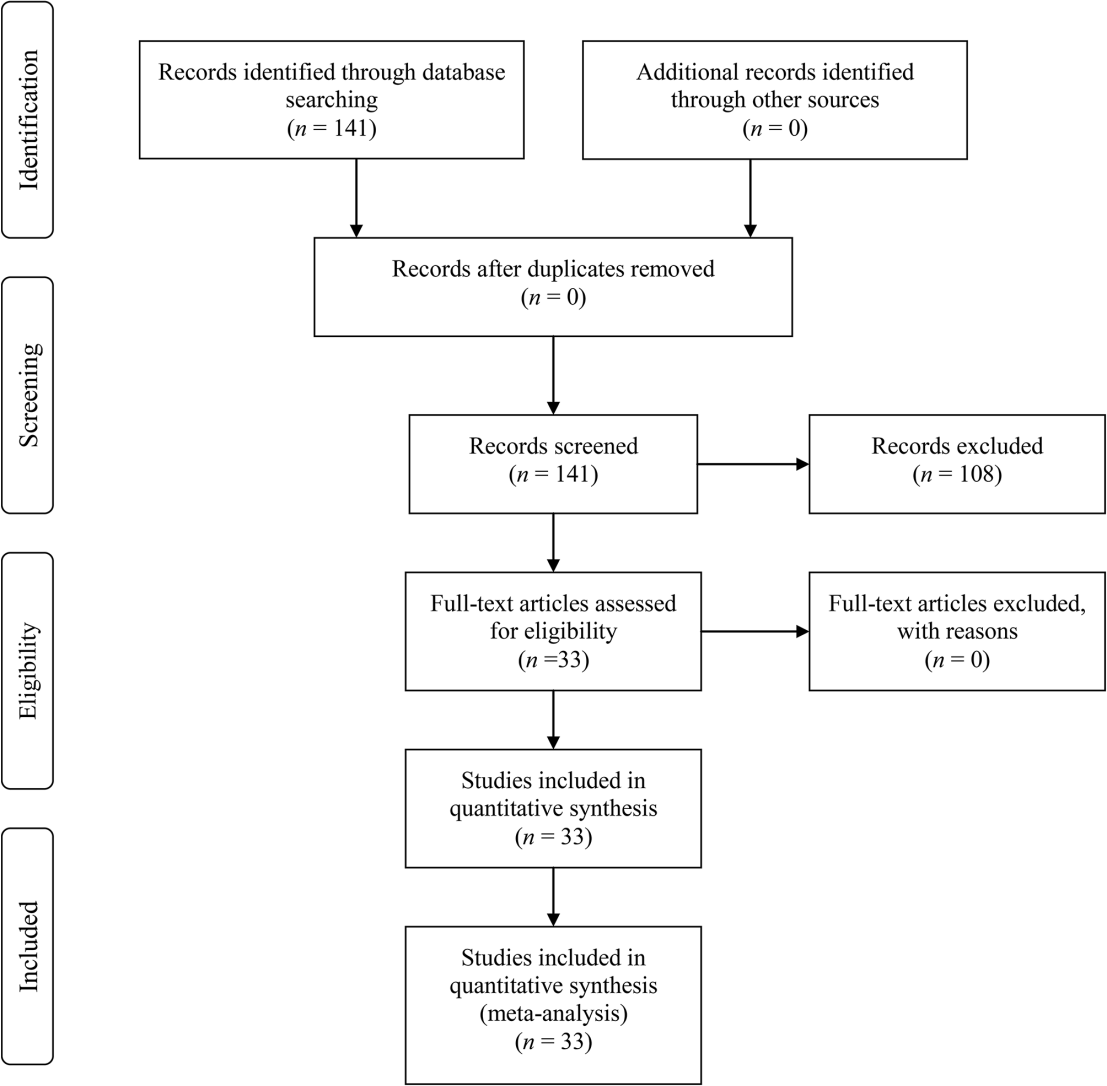
The flow diagram of PRISMA. Note PRISMA = preferred reporting items for systematic reviews and meta-analyses

This study aims to integrate the relationships among emotional labor, job burnout, and emotional intelligence. Using integrative analysis as the research method for literature collection, it synthesizes samples and data related to the relevant variables, rather than employing a quantitative research questionnaire survey method. A substantial amount of data was gathered from previous relevant literature through electronic databases, including “Airiti Library,” “Taiwan Master’s and Doctoral Thesis Knowledge Value-Added System,” “National Library Journal Information Network,” “Web of Science,” and “Proquest.” The keywords “emotional labor,” “job burnout,” and “emotional intelligence” were utilized for extensive research collection. The collected data must include the correlation coefficients of the three research variables and their pairwise relationships, including studies from research journals and master’s and doctoral theses. To avoid duplication in calculations, if a master’s or doctoral thesis has been submitted to a journal, the research journal will be prioritized as the main source of data collection. The selection of research samples was based on the following criteria (Fig. 1):

- Literature data collection was filtered by quantitative research methodologies, requiring compliance with questionnaire survey methods and excluding qualitative research literature.
- The literature collection primarily utilized electronic databases, ensuring access to full-text articles and relevant coefficients.
- The collection of literature from research journals and master’s and doctoral theses focused on articles published before October 27, 2024.
- Considering cultural similarities, samples were collected from Taiwan and other Asian regions.
- If the collected literature shows significant discrepancies in results, the research samples do not meet the standards of this study, or if the questionnaire response rates are too low, such data will not be included in the study’s data collection database.

This study collected data from journal articles and master’s and doctoral theses published before October 27, 2024. Using electronic databases, keywords such as emotional labor, job burnout, and emotional intelligence were employed to search for a total of 53 journal articles and 88 theses. After excluding studies that did not include the relationships between emotional labor, job burnout, and emotional intelligence, as well as qualitative research, some journal articles and theses that lacked relevant coefficients were also removed. The final literature collection comprised 28 journal articles and 5 theses. The final sample of included literature consisted of 28 journal articles and 5 master’s theses or doctoral dissertations, totaling 33 empirical studies used as the sample for analysis in this research.

**Table 1:** Summary of sources for integrative analysis report on emotional labor, job burnout, and emotional intelligence

Data Source	Emotional labor &job burnout		Emotional intelligence & job burnout		Emotional intelligence & emotional labor	
	Collected Count	Selected Count	Collected Count	Selected Count	Collected Count	Selected Count
Research Journals	31	13	13	6	9	9
Master’s & Doctoral Theses	72	0	9	2	7	3
Total	103	13	22	8	16	12

### Data analyses

The present study employed a MASEM model. First, an MA was conducted to collect the relevant literature and analyze the corresponding data, with the objective of exploring the relationships between variable pairs. Subsequently, an integration method was applied to analyze the research sample and data. Effect size was used as a quantitative measure to identify objective general conclusions from the collected literature. The statistical software Comprehensive Meta-Analysis (CMA) was used to convert the correlation coefficients between each variable pair (EL–JB, EL–EI, and JB–EI) into equivalent effect sizes to clarify the correlations between the examined variables and create referential plots for further analyses. A test of homogeneity was also conducted to determine how journal articles differed from master’s theses and doctoral dissertations. The relationships between EL, JB, and EI were explained to verify the relationships between each variable pair and among all three variables. Subsequently, SEM was employed to validate the model fit. Being a method that combines factor analysis and path analysis, SEM can be employed to validate all hypotheses and provide exploratory suggestions for validating model fit.

## Results

### Relationship between emotional labor and job burnout

After a literature screening was conducted, 13 research samples were included. The homogeneity test results revealed a Q value of 321.889, which was statistically significant (*p* < 0.001) and indicated the presence of heterogeneity in the present study (i.e., the null hypothesis of homogeneity was rejected). Generally, in heterogeneous studies, random-effects models are used to perform interpretations. However, the small sample size of the present study did not allow for a meaningful interpretation of the results obtained from random-effects models. Therefore, a fixed-effects model was adopted. An I^2^ value of 96.272 was obtained, indicating a high degree of heterogeneity. Therefore, the present study explained a high level of the heterogeneity, with the obtained mean explaining 96.272% of the variance. The effect size between EL and JB was 0.246, indicating a low degree of correlation. The corresponding 95% confidence interval (CI) ranged from 0.220 to 0.271. The estimated effect size was converted into a Z value of 18.236, which was statistically significant (*p* < 0.001). Thus, the analysis results indicated a positive correlation between EL and JB (Table 1).

**Fig. 2:**
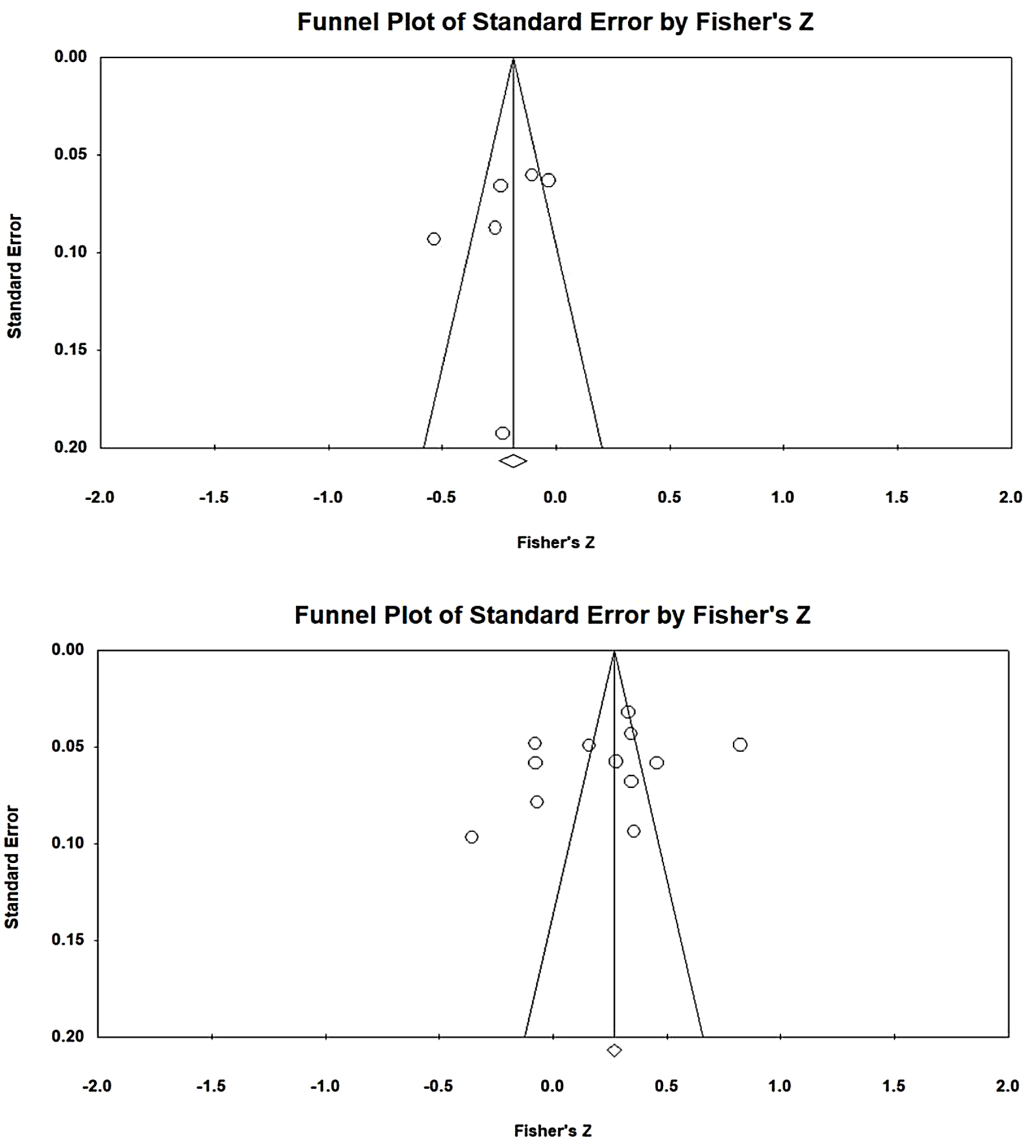
Funnel plot of EL and JB analysis results

To avoid the overestimation of effect sizes due to publication bias, a publication bias analysis of the included samples was conducted. First, a funnel plot was created for examination. Figure 2 presents the funnel plot for EL and JB, revealing that all 13 included articles were distributed on both sides of the funnel and formed a mostly symmetrical shape. No evidence of publication bias was found. To examine publication bias, the fail-safe N for the relationship between EL and JB was calculated and determined to be 922, indicating that 922 additional studies with nonsignificant results pertaining to EL and JB were required to overturn the findings of the present study. The tolerance interval (5 K + 10, where K represents the total number of samples included in the MA) for the present study was 75 articles. According to Rosenthal, when the fail-safe N exceeds the tolerance interval, unpublished, nonsignificant, and undiscovered studies not included in an MA do not affect its results [40]. The fail-safe N in the present study was greater than the tolerance interval; thus, the present study had no publication bias with respect to relationship between EL and JB (Table 2).

**Table 2:** Meta-analysis results for relationships between variables

Variable	*N*	K	dw		95% CI		Z value	Test of homogeneity		Fail Safe *N*
			δ	SDd	Lower limit	Upper limit		Q value	I^2^	
EL and JB	13	5329	0.246	0.015	0.220	0.271	18.236^***^	321.889^***^	86.272	922
EL and EI	12	2008	0.288	0.022	0.200	0.257	15.183^***^	463.054^***^	97.625	701
EI and JB	8	4304	− 0.0.189	0.013	-0.231	-0.146	-8.503^**^	117.107^***^	84.023	99

^***p<0.001^

### Relationship between emotional labor and emotional intelligence

**Fig. 3:**
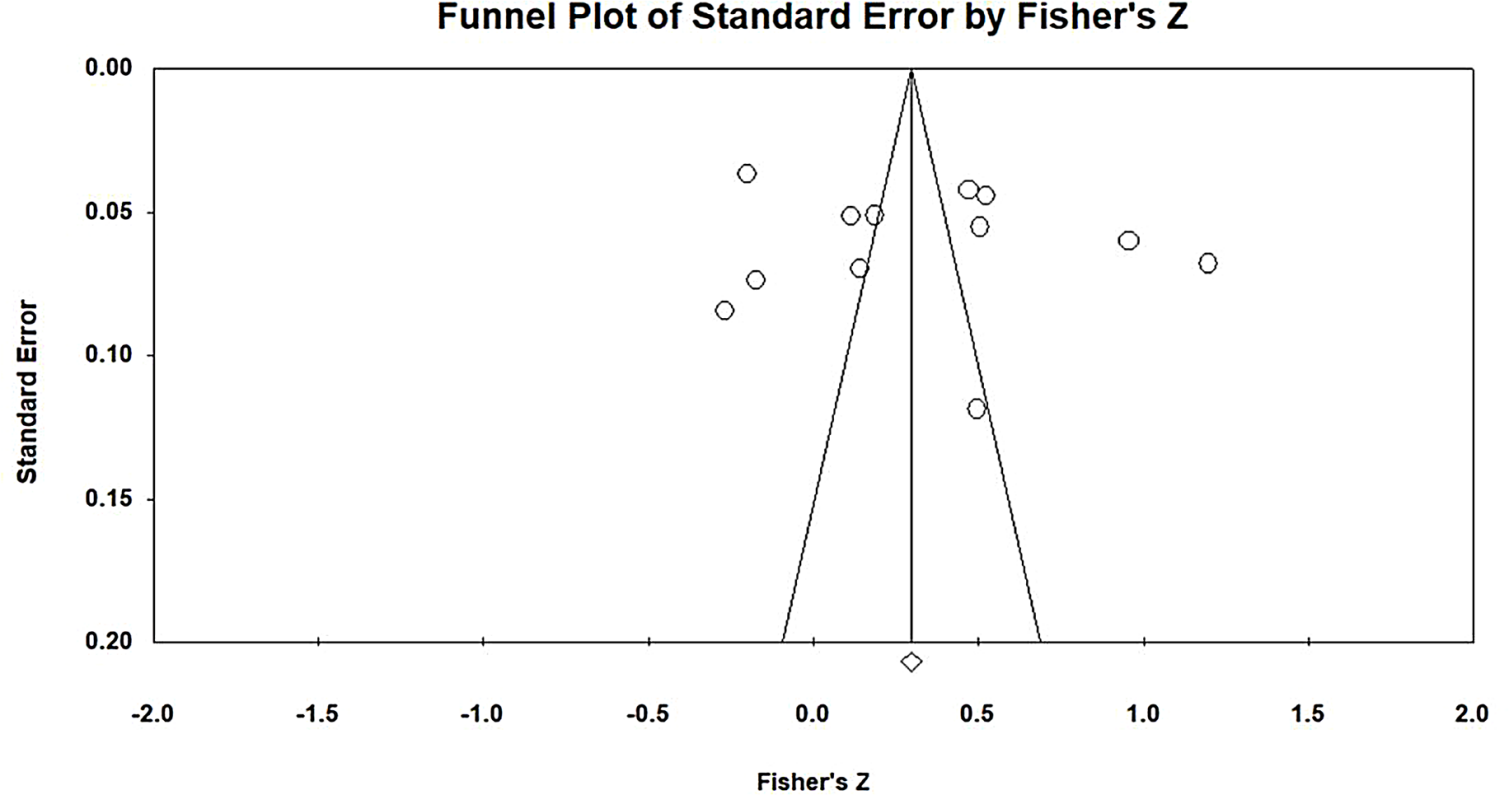
Funnel plot of EI and EL analysis results

After a literature screening was conducted, a total of 12 research samples were included. The homogeneity test revealed a Q value of 643.504, which was statistically significant (*p* < 0.001) and indicated the presence of heterogeneity in the present study (i.e., the null hypothesis of homogeneity was rejected). In heterogeneous studies, random-effects models should be used for interpretation. However, the sample size was too small to yield meaningful interpretation of the results of random-effects models. Therefore, a fixed-effects model was adopted. An I^2^ value of 97.624 was obtained, indicating a high degree of heterogeneity. Therefore, the present study explained a high level of heterogeneity, with the obtained mean explaining 97.624% of the variance. The effect size between EI and JB was 0.288, indicating a low degree of correlation. The corresponding 95% CI ranged from 0.200 to 0.257. The estimated effect size was converted into a Z value of 15.183, which was statistically significant (*p* < 0.001). Thus, the analysis results indicated a positive correlation between EI and EL. The corresponding funnel plot revealed that all 12 included articles were distributed on both sides of the funnel and formed a mostly symmetrical shape (Fig. 3). No evidence of publication bias was found. The fail-safe N for the relationship between EI and EL was 701, indicating that 701 additional studies with nonsignificant results pertaining to EI and EL were required to overturn the findings of the present study. Given that the tolerance interval was 70 articles, the present study had no publication bias with respect to the relationship between EI and EL.

### Relationship between emotional labor and job burnout

**Fig. 4:**
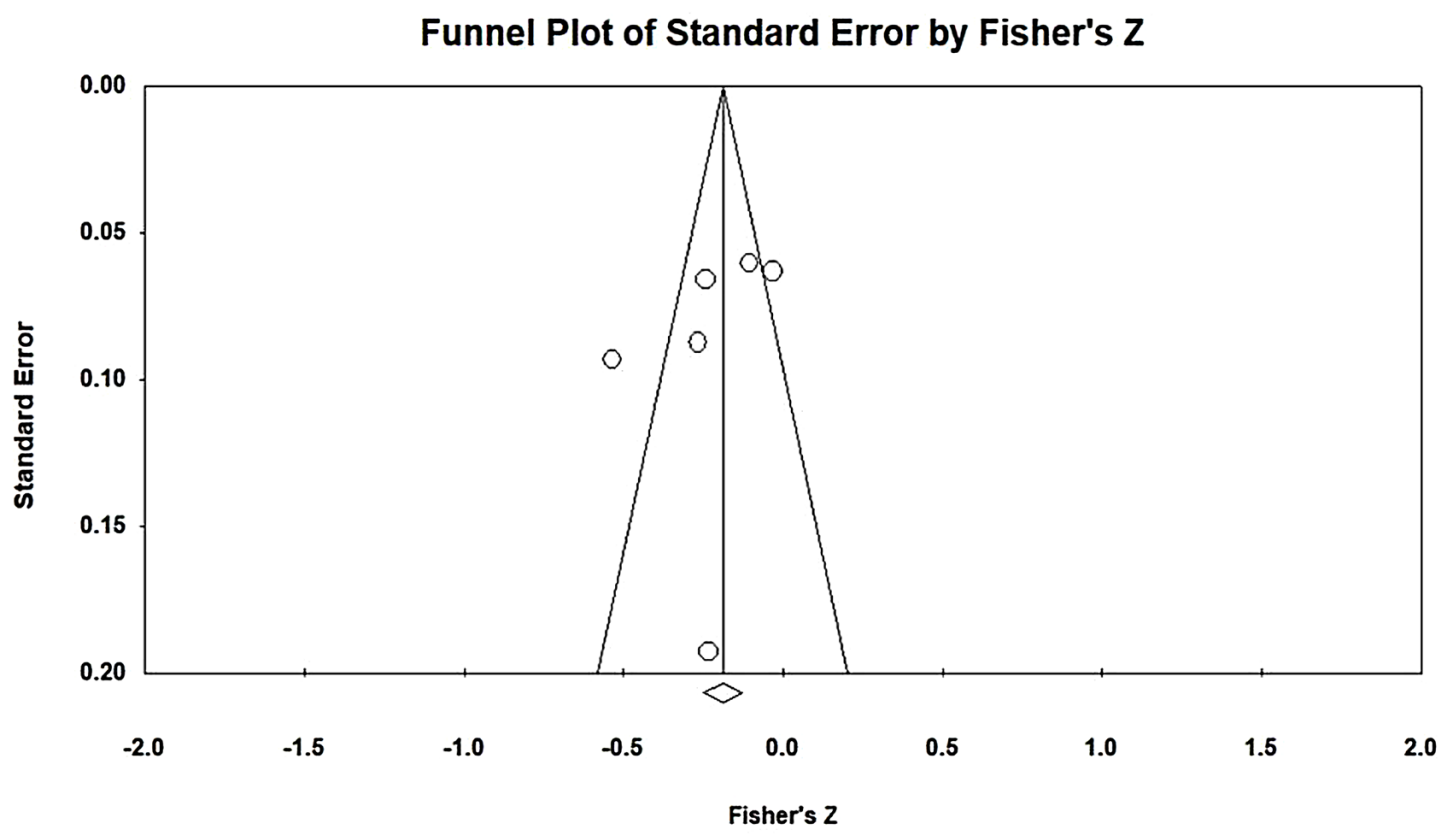
Funnel plot of EI and JB analysis results

After a literature screening was conducted, a total of 8 research samples were included. The homogeneity test result revealed a Q value of 117.107, which was statistically significant (*p* < 0.001) and indicated the presence of heterogeneity in the present study (i.e., the null hypothesis of homogeneity was rejected). In heterogeneous studies, random-effects models should be used for interpretation. However, the sample size was too small to yield meaningful interpretation of the results of random-effects models. Therefore, a fixed-effects model was adopted. An I^2^ value of 94.023 was obtained, indicating a high degree of heterogeneity. Therefore, the present study explained a high level of heterogeneity, with the obtained mean explaining 94.023% of the variance. The effect size between EI and JB was − 0.189, indicating a low degree of correlation. The corresponding 95% CI ranged from − 0.231 to − 0.146. The estimated effect size was converted into a Z value of − 8.503, which was statistically significant (*p* < 0.001). Thus, the analysis results indicated a negative correlation between EI and JB. The corresponding funnel plot reveals that all the 8 included articles were distributed on both sides of the funnel and formed a mostly symmetrical shape (Fig. 4). No evidence of publication bias was found. The fail-safe N for the relationship between EI and JB was 99, indicating that 99 additional studies with nonsignificant results pertaining to EI and JB were required to overturn the findings of the present study. Given that the tolerance interval was 50 articles, the present study had no publication bias with respect to the relationship between EI and JB.

### Validation of the theoretical models of emotional labor, job burnout, and emotional intelligence

#### Correlation matrices, harmonic mean, and reliability between emotional labor, job burnout, and emotional intelligence

Through the MA, the present study obtained a correlation coefficient matrix for the relationships between EI, EL, and JB (Table 3), and the matrix was used to validate the subsequent SEM. Because the number of samples varies between different variable pairs (i.e., EI, El, and JB in Table 3), to prevent the differences in sample sizes from affecting the results and to generate non-positive-definite problems, the present study followed the recommendations of Viswesvaran and Ones and converted the samples sizes (for the studies that examined the correlations between each variable pair) into harmonic means to obtain the overall sample size [41]. The calculated harmonic mean of 3,268 was used for SEM validation (Table 4).

**Table 3:** Correlation coefficient matrix for relationships between EL, JB, and EI

Variable	EI	EL	JB
EI	1.000		
EL	0.228	1.000	
JB	-0.189	0.246	1.000

**Table 4:** Sample sizes of studies that examined correlations between EL, JB, and EI

Variable	EI	EL
EL	4304	
JB	2008	5329

Table 5 presents the corrected reliability results for the included studies with respect to each variable. The reliability values were used to calculate the mean reliability, residual, and root mean reliability of each study, and they were used in the SEM process.

**Table 5:** Reliability of included studies with respect to EL, JB, and EI

Author	Year	EL	JB	EI
Lin et al.	2015		0.84	
Liu et al.	2016	0.939	0.949	0.956
Liu and Hsieh	2016	0.857	0.895	
Chen et al.	2017	0.882	0.874	
Chen and Wu	2019	0.901	0.92	
Angeli et al.	2015			0.86
Aeeun Jeon	2016		0.782	
Liu et al.	2018		0.79	0.95
Kwon et al.	2021	0.79	0.94	
Kim	2020		0.81	
Lee and Ji	2018		0.75	
Hung and Liu	2011		0.887	0.93
Hung	2008		0.834	0.873
Hsieh et al.	2008	0.59	0.87	
Ponniah Ramana et al.	2016		0.95	0.87
Parviz et al.	2023		0.71	0.75
Lin	2009	0.885		0.887
Wong	2017	0.893	0.913	0.897
Kamassi et al.	2019			0.919
Aisha et al.	2023	0.93		0.92
Mean reliability		0.852	0.850	0.890
Residual		0.148	0.150	0.110
Root mean reliability		0.923	0.922	0.943

#### Validation of theories regarding the relationships between emotional labor, job burnout, and emotional intelligence

## Basic fit standards

Figure 4 presents that the parameter estimates for the relationships between EI and JB, EI and EL, and EL and JB, which are all positive and less than 1. The *p*-values were all significant, suggesting that the parameter estimation values conformed to a basic fit. The factor loadings for EI, EL, and JB were 0.943, 0.923, and 0.922, respectively, which slightly exceeded the factor loading level of 0.95 recommended by Bagozzi and Yi; however, these values were within the acceptable range [42].

According to the table summarizing the parameter estimates for latent variable errors, the error estimation values were positive, and the standard errors ranged between 0.026 and 0.029; this range was within the moderate range and conformed to the basic fit for moderate standard errors as proposed by Bagozzi and Yi [42]. These results revealed that the parameters of the proposed model met the criteria for acceptable parameter values, indicating a high-quality model.

In summary, only the factor loading for EI (0.951) was slightly higher than the indicator norm of 0.95. The model error variance results did not reveal any negative value. That is, all values were positive and significant. The absolute value of the correlation coefficients between estimation parameters did not approach 1, and the standard error was not excessively high, satisfying the standard for basic fit. Thus, the proposed theoretical model was acceptable.

## Overall model fit

The goodness of fit index (GFI) value for the present study was 1 (a GFI value that is closer to 1 indicates a more favorable fit), which was higher than the recommended threshold of 0.9 and indicated an acceptable fit. Both the root mean square residual (RMR) and standardized root mean square residual (SRMR) were 0, that is, less than the suggested thresholds of 0.05 and 0.08, respectively; these results indicate that the fit criteria were satisfied. When SRMR equals 0, a perfect fit is achieved. Therefore, the absolute fit indices for the present study met the fit standards, indicating that the model of the present study exhibited a favorable fit. For the incremental fit indices, the normed fit index (NFI), incremental fit index (IFI), and comparative fit index (CFI) values were all 1, exceeding the fit criterion of 0.9. These values indicated that the incremental fit indices met the established criterion, demonstrating the favorable fit of the proposed model. For parsimony fit indices, the parsimonious normed fit index (PNFI) for the present study was 0, which did not exceed the threshold of 0.05, showing compliance with the fit index criterion. However, the Akaike information criterion (AIC), Bayesian information criterion (BIC), and expected cross-validation index (ECVI) are competitive model fit indices, and they could not be applied in the present study because the proposed model was not competitive. Therefore, no further discussion or comparison of AIC, BIC, and ECVI values are conducted in the present study.

In summary, the overall fit analysis indicated that nearly all the fit indices met their respective fit criteria. The absolute fit indices and incremental fit indices met the fit criteria. Among the parsimony fit indices, the PNFI was the only index that did not meet the recommended standard. The results for the AIC, BIC, and ECVI were used in competitive models; thus, they were unsuitable for the proposed model in this study. In the present study, only a single parsimony fit index did not meet the recommended standard. These results indicated that the theoretical model fit was favorable such that a subsequent exploration could be conducted.

## Fit of internal structure model

The individual reliability values for EL, JB, and EI were higher than 0.5; that is, they all satisfied the recommended standard. The composite reliability was greater than 0.6, and the mean variance extracted of all variables was greater than 0.5; that is, these values all satisfied the recommended standard. These results indicate that the proposed model had a favorable internal structure model fit. The standardized residual test revealed that all values were less than 1.96, indicating that the internal structure model fit met the recommended standard and was favorable.

### Moderating Effect of EI on Relationship between Emotional Labor and Job Burnout

To validate moderating effects, bootstrapping was conducted with a 95% interval and 1000 iterations. Table 6 presents the results for the moderating effect validation conducted using bootstrapping. Error modification percentage bootstrapping and percentage bootstrapping were performed to examine the related effects. The 95% CI did not include a zero value, and the *p*-value was significant, confirming the moderating effect of EI. These results indicated that EI moderated the relationship between EL and JB.

**Table 6:** Bootstrapping validation of moderating effect

Parameter	Method	Estimation	Lower limit	Upper limit	*p*-value
EL→EI→JB	Error modification percentage bootstrapping	-0.082	-0.101	-0.0966	0.001
	Percentage bootstrapping	-0.082	-0.100	-0.065	0.002

EL was verified to have a total effect, direct effect, and indirect effect on JB. The total effect of two variables in three variables was significant. The total effect size of EL on EI was 0.262, indicating a direct effect. The total effect size of EI on JB was − 0.315, indicating a direct effect and supporting the hypotheses proposed in the present study. No indirect effect was identified with respect to the relationships between EL and EI and between EI and JB. The total effect size of EL on JB was 0.289, which was the sum of the direct effect and indirect effect sizes (0.371 − 0.082). This result signified that EL affected JB through EI and that EI moderated the relationship between EL and JB, supporting Hypothesis 4.

## Discussion and suggestions

### Discussion

#### Relationship between emotional labor and job burnout

The results of the present study revealed a significant and positive relationship between EL and JB, indicating that when employees experienced higher levels of EL, they also perceived an increase in JB. The present study assumed that EL is a type of job requirement, where employees are subjected to the intangible or tangible emotion norms of an organization and expected to provide services through face-to-face or vocal interactions. In this situation, their emotions are subject to organizational management. Furthermore, the emotions of employees can be cultivated through training such that they can display specific emotional expressions; this process is regarded as an aspect of EL [1]. Depending on the occupations that are involved, an organization may require its employees to express specific emotions or service attitudes toward customers. If employees effectively display the positive emotions required of them, they can feel a sense of accomplishment through their emotional influence on customers. Therefore, EL is regarded as a performance indicator of service quality and overall impression, and it is closely related to the job outcomes of employees [43]. However, when employees self-regulate their emotions, they often encounter constraints and need to display professionalism while hiding their true emotions. This process of excessive emotional suppression results in an inconsistency between internal thoughts and external expressions, and it can lead to the development of several types of JB including emotional exhaustion, depersonalization, and reduced sense of accomplishment. The faking of emotions depletes the energy of an employee and causes them to experience frustration, and it is also a main factor that contributes to work dissatisfaction [12, 44]. According to conservation of resources theory, the level of an employee’s EL affects their emotional resources during emotional transitions. The dissonance between genuine emotions and emotion norms can lead to the development of JB.

#### Relationship between emotional intelligence and emotional labor

The results of the present study revealed a significant and positive relationship between EI and JB, indicating that when employees possess a higher level of EI, their adoption of EL coping behavior and their generation and perception of EL increase. On the basis of conservation of resources theory, the present study regarded EI as the internal emotional resources of an individual employee. EI encompasses the ability to perceive and evaluate one’s emotions and those of others and the ability to regulate and apply emotions. Thus, when employees have higher EI, they are more likely to adjust their emotional expressions through deep acting to align with their internalized emotions and organizational requirements when they engage in EL at work. This alignment allows them to gain recognition and psychological support, reduce the depletion of their emotional resources, and reinforce these resources [21, 45]. Moreover, the emotional demands of an organization can lead to the development of EL among its employees, and their individual adaptation is dependent on their EI. Employees with higher EI exhibit a higher sense of environmental safety and are more skilled at social interactions. They can effectively identify their genuine inner emotions and establish a connection between EL and their inner emotions. They are also more likely to engage in deep acting as an adaptive mechanism for managing their internal emotions. These individuals have greater EL loading levels. Thus, individuals with higher EI are regarded as more suitable candidates for undertaking EL [32, 46].

#### Relationship between emotional labor and job burnout

The results of the present study confirmed that the proposed hypotheses are consistent with the findings of other studies. Several Taiwan-based studies reported that a significant and negative relationship existed between the EI and JB of nursing and rehabilitation professionals. Although these professions are associated with emotions related to safety, care, and joy, they must frequently confront the illness and mortality of patients. Thus, individuals with higher EI can regulate their emotions and mitigate negative emotions that cause psychological discomfort. Individuals with higher EI are more empathetic toward the emotions of others and are more adept at understanding and managing their own emotions and those of others. Thus, they are more likely to exhibit rational behavior, experience a sense of accomplishment, and experience less frustration caused by work and interpersonal interactions [47, 48]. Liao and Song examined mass rapid transit drivers with high job stressors, and their results indicated that the drivers with higher EI were more adept at using emotional information to guide appropriate work behavior, enabling them to effectively convert their job stress into opportunities for professional development [28]. Moreover, individuals with higher EI exhibit higher emotional tolerance. They adopt positive and proactive attitudes when faced with setbacks at work and do not avoid job responsibilities; instead, they inspire themselves to achieve personal accomplishments, thereby reducing their JB. Similar findings have also been reported by studies conducted outside of Taiwan. Studies that examined teachers have discovered that the application of EI enabled teachers to effectively manage their job frustrations, implement emotional control, maintain harmonious relationships with their colleagues, achieve enhanced self-awareness, and improve their job performance. These studies highlighted that accomplishments and goal attainment are not only dependent on knowledge, skills, and experience, but also on the ability to manage one’s emotions. Successfully understanding oneself and others lead to improved outcomes and consolidates the importance of emotions and the negative relationship between EI and JB [49].

#### Relationships between emotional labor, job burnout, and emotional intelligence

The present study proposed a theoretical model and several hypotheses regarding EL, JB, and EI variables. On the basis of conservation of resources theory, the present study examined employee emotions as a job resource. Accordingly, when employees engaged in EL, having higher EI helped them adopt deep acting strategies to enhance their EL loading levels. The job requirements-resources model revealed that employees with higher EI were more adept at understanding others and regulating their own emotions, enabling them to adapt quickly to the emotion norms of their organizations and to alleviate the job stress and burnout caused by emotional dissonance. In other words, the EL of employees was affected by their level of EI, which in turn affected the reduction of JB. EI moderated the relationship between EL and JB.

Individuals with high EL who experienced a discrepancy between emotional requirements and their own emotions were more likely to have a negative sense of JB. However, when employees engaged in EL, those with emotional awareness and the ability to perform effective emotion transitions experienced reduced JB as a result of regulated deep acting [12]. Both studies conducted within and outside of Taiwan have extensively discussed the relationship between high EL jobs, EI, and JB. Most studies have employed conservation of resources theory, which regards employee emotions as a type of job resource. If emotions are regarded as job requirements, they may promote emotional labor performance and mitigate the level of JB [33].

**Fig. 5:**
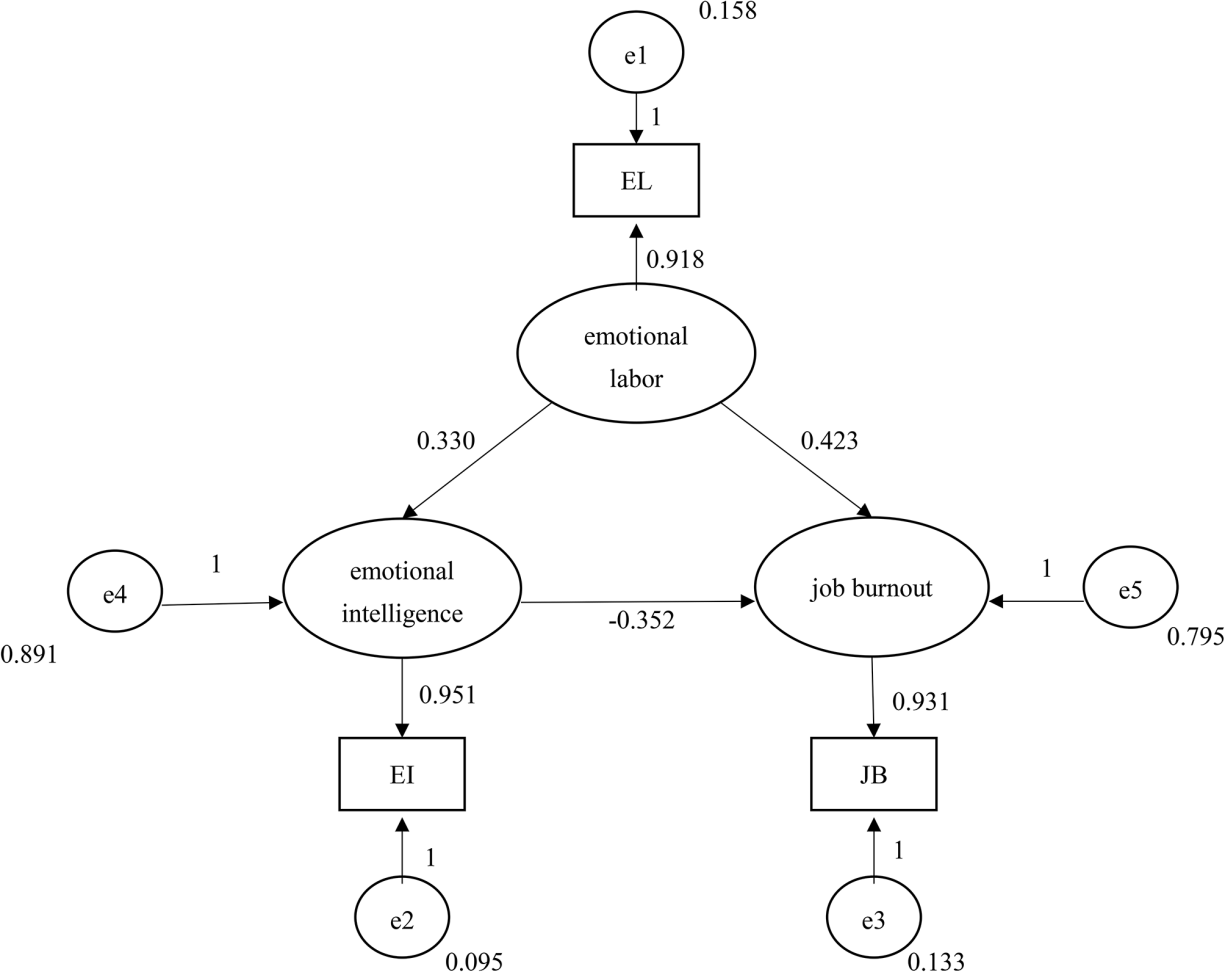
Path chart and standardized parameter estimates of proposed theoretical model

The overall fit index results presented in the model fit summary table indicate a favorable fit between the proposed theoretical model and the collected data (Fig. 5). Nearly all the fit index results, including the absolute and incremental fit index results, met the evaluation criteria. The PNFI was the only index for which the results were slightly less than the recommended standard of 0.05. The results indicated that the basic model fit, overall model fit, and internal structure model fit of the proposed model were all satisfactory. Additionally, in the analysis of the moderating effects of EI, bootstrapping with 1000 iterations was performed with the CI being set to 95%. The results did not include a zero value, and the total effect of EL on JB was 0.307, which was equivalent to the sum of the direct effect (0.423) and the indirect effect (− 0.116) (0.423 − 0.116).

### Suggestions

#### Management implications

The results revealed a significant and positive relationship between EL and JB. Therefore, in terms of management practices, organizations should establish emotion-related rules to meet customer service expectations with respect to emotional expression. They should also examine other factors that may affect employees, such as their work environment, the target recipients of a service, and the occurrence of unexpected events. These measures enable organizations to mitigate the negative effects of EL on the development of JB in their employees. For employee training, organizations should focus on teaching deep acting strategies for emotional expression. In contrast to masking or surface acting, this approach can encourage employees to naturally express specific emotions that can positively influence their colleagues and customers. It can also substantially reduce the development of JB among employees at work.

The results indicated a positive relationship between EI and EL. This relationship encompasses the adoption of deep acting strategies by employees and an increase in their level of EL loading. Therefore, businesses should not only consider basic requirements during the selection process but also incorporate an EI assessment or observe the ability of candidates to express themselves and monitor their surroundings during conversations. To facilitate talent management, organizations must consider EI in terms of personnel promotion and relocation decisions. A manager must not only set emotion norms but also understand the challenges that their employees face and the process of emotional transition that they undergo during EL. Employees with higher EI can empathize with people in these situations and offer support to other employees who need it. This method can effectively propagate an organization’s emotional demands among its personnel.

The results revealed a negative relationship between EI and JB, indicating that employees with higher EI generate less JB and perceive lower levels of JB. Therefore, organizations should consider incorporating EI as a predictor of employee performance and burnout during their selection process. Additionally, organizations can help employees enhance their EI through education and training, thereby cultivating their ability to perceive the emotions of others and anticipate the needs of their customers before any requests are made. This measure can foster enthusiasm and a sense of accomplishment among employees at work. Furthermore, implementing job rotation and task transitions can help employees cultivate their EI and reduce their JB through the accumulation of practical experience.

The present study verified the significant moderating effect of EI on the relationship between EL and JB. When employees have higher EI, they can more effectively adapt to the emotion norms within their organization. They can also transform their EL into genuine experiences and engage in deep acting to mitigate JB. In other words, an employee’s level of EI can affect their reduction of JB resulting from EL. Therefore, organizations should use EI as a criterion for assessing the self-awareness and personal emotional management of candidates during the selection process. EI can predict how candidates adapt to EL in future job roles and their ability to cope with work-related stress. For candidates who demonstrate high levels of professional competency and the potential to improve their EI, an organization should provide training programs that incorporate EI training. These programs should incorporate experiential learning to enhance the emotional regulation and resilience of employees in the face of setbacks. When human resources teams identify negative work outcomes caused by EL or EI-related JB, they can use their organizations’ internal resources to implement job rotation or job redesign. Practical experiences can be leveraged to cultivate the EI of employees. Additionally, providing counseling and care for employees can contribute to talent retention. If an organization can promote methods that emphasize positive emotions to guide employees to accept challenges with a positive attitude, the EI levels of the organization’s teams can be increased, and a positive atmosphere can be created. Subsequently, the JB caused by EL can be reduced, thereby reducing turnover intention.

In many collectivist societies in Asia, culture often emphasizes harmony, respect for hierarchy, and group cohesion. These values may influence how emotional labor is performed and perceived. Compared to Western contexts, the impact of cultural values on the dynamics of emotional labor is more pronounced in Asia. Many Asian countries have fast-paced and high-pressure work environments, particularly in sectors such as education, healthcare, and customer service. These environments create unique sources of stress that exacerbate job burnout. It is important to delve into how cultural expectations regarding diligence, long working hours, and loyalty to employers contribute to heightened levels of job burnout in the Asian region.

The influence of emotional intelligence (EI) in Asian organizations may also differ, as social harmony and indirect communication styles hold significant meaning in these cultures. High emotional intelligence can enhance employees’ adaptability to cultural and organizational norms, especially in managing emotions within hierarchical relationships and customer interactions. The application of the Conservation of Resources (COR) theory in collectivist cultures may vary, as employees in these cultures often face greater pressures to achieve collective goals, making emotional labor more demanding. Therefore, it is crucial to emphasize how emotional intelligence helps employees in Asia alleviate stress and adapt to job requirements within unique social and organizational structures.

#### Future suggestions

EL, JB, and EI have been studied over a long period of time. Scholars have proposed various definitions and explored various perspectives relating to these constructs. For example, EL is often classified as surface acting and deep acting, but some scholars have argued that an individual’s genuine emotions should also be regarded as form of emotional display and be established as a third category of EL. Scholars have also proposed alternative definitions other than the aforementioned categories. However, in the present MA study, the analysis focused on exploring the correlations among variables by examining a single variable without separately integrating and validating individual subdimensions through SEM. Given the integrity of the dimensions, future studies should focus on extending the topics explored in the present study; this can be achieved by exploring the subdimensions of each variable to clarify in-depth research conceptions and further verifying the consistency of the relationships between single variables and subdimension variables.

The literature review revealed that a strong connection exists between EL, JB, and EI in both academic and practical contexts. The related topics, such as organizational culture, leadership style, and the work stress that influences employee development, are frequently discussed in organizational settings. Various studies have explored work outcome–related topics such as turnover intention, retention willingness, and job satisfaction. Therefore, future studies can use the findings of the present study as a theoretical foundation for exploring other moderating variables and their corresponding effect sizes. Given that the present study focused on EL, JB, and EI, which are individual-level variables, future studies should examine organizational-level or group-level variables to expand the research on EL, JB, and EI and facilitate the development of detailed recommendations for practical measures.

The research methods employed in the present study combined MA and SEM. The initial data collection step involved identifying studies that investigated the variables of interest and conducting data analysis through MA. Subsequently, on the basis of the correlations between each variable pair, the fit of the SEM was validated. This integrated approach focused on effect sizes, which is a quantitative measure. However, to obtain a more comprehensive understanding of the relationships examined in the present study, future studies should collect qualitative data through methods such as interviews, observations, or other methods used in the relevant qualitative studies. Through the application of various research methods in conjunction with quantitative analysis, the reliability of the validation results can be enhanced.

To research EL, JB, and EI, researchers have employed various definitions and scales depending on the dimensions of the variables that were examined. For instance, to measure EL, some scholars have defined it on the basis of its acting aspect, whereas others have classified EL on the basis of interactions or diversity-related dimensions. Similarly, for EI, researchers have used scales that assess personal emotions and the emotions of others and the ability of an individual to evaluate and apply emotions. Researchers may be inclined to adopt scales with a higher reliability for evaluation, and they often prefer scales that were developed during the early stage of variable content development. Therefore, given the need to align past research with the current context, researchers should compare newer and older scales or prioritize the analysis of scales developed in more recent years. Furthermore, study samples vary across studies. Thus, to account for the potential influence of occupational differences in job content on research outcomes, researchers should consider obtaining consistent samples or focusing on specific professions to facilitate data integration.

The present study considered the uniformity of regions and the similarity of cultures in data collection by selecting studies from Taiwan and the Asian region. Future studies should consider cultural differences and employ a cross-cultural research design to collect data from both Taiwan and international sources. In addition to studies from the Asian region, studies from Western countries can be included. Furthermore, expanding literature sources to include foreign dissertations and theses can increase the comprehensiveness of the relevant research and enable the extension of findings to various countries and industries, thereby providing a more comprehensive interpretation with major managerial implications.

## Electronic supplementary material

Below is the link to the electronic supplementary material.

Supplementary Material 1

## Data Availability

The datasets analysed for the current study are available from the corresponding author upon reasonable request.

## Abbreviations

EL: Emotional Labor
JB: Job Burnout
EI: Emotional Intelligence
SEM: Structural Equation Modeling
MASEM: Meta-Analysis Structural Equation Modeling
MBI: Maslach Burnout Inventory
Mas: Meta-Analyses
CMA: Comprehensive Meta-Analysis
GFI: Goodness Of Fit Index
RMR: Root Mean Square Residual
SRMR: Standardized Root Mean Square Residual
NFI: Normed Fit Index
IFI: Incremental Fit Index
CFI: Comparative Fit Index
PNFI: Parsimonious Normed Fit Index
AIC: Akaike Information Criterion
BIC: Bayesian Information Criterion
ECVI: Expected Cross-Validation Index
COR: Conservation Of Resources
